# Inpatient Monitoring of Decompensated Heart Failure: What Is Needed?

**DOI:** 10.1007/s11897-017-0352-x

**Published:** 2017-08-12

**Authors:** Danish Ali, Prithwish Banerjee

**Affiliations:** 1 Department of Cardiology, University Hospital Coventry and Warwickshire, Clifford Bridge Road, Coventry, CV2 2DX UK; 2 Faculty of Health & Life Sciences, Coventry University, Coventry, UK

**Keywords:** Decompensated heart failure, Monitoring

## Abstract

**Purpose of Review:**

Acute decompensated heart failure is a serious and common condition where close monitoring of symptoms, vital signs, haemodynamic and other markers are needed after the patient is admitted to hospital as the in-hospital outcome is poor. This review focuses on advances in the assessment and monitoring of these patients.

**Recent Findings:**

The adoption of the CHAMP acronym to identify precipitating factors and of the classification using wet-warm, wet-cold, dry-warm and dry-cold categories is an improvement regarding assessment.

**Summary:**

Although the outcome of acute decompensated heart failure has remained poor with no new treatments found for a number of years, a structured approach to assessment and monitoring is now available.

## Introduction

Acute decompensated heart failure (ADHF) is a fatal condition, in which there is rapid onset or deterioration of symptoms and/or signs of heart failure. It is the leading cause of hospital admission of over the age of 65 in the UK and the USA. (https://www.nice.org.uk/guidance/cg187/chapter/Introduction). Worldwide, there are at least 20 million patients from suffering from heart failure (HF) and over 1 million HF hospitalizations per year occur in the USA alone [1]. Admission with ADHF is associated with a high 1-year mortality of approximately 30% [2]. Patients with ADHF require urgent medical assessment and treatment, and inpatient admission itself carries an in-hospital mortality (especially in the elderly) of approximately 13% [3, 4]. Whilst over the past two decades, the stable chronic HF patients have experienced significant improvement in their prognosis with the discovery of new HF drugs; the same cannot be said about ADHF patients [5]. Therefore, thorough inpatient monitoring and management for this large patient group is needed. This article focuses mainly on the in-hospital monitoring following an admission with ADHF; treatment is dealt with in separate articles.

## Classification

**Fig. 1:**
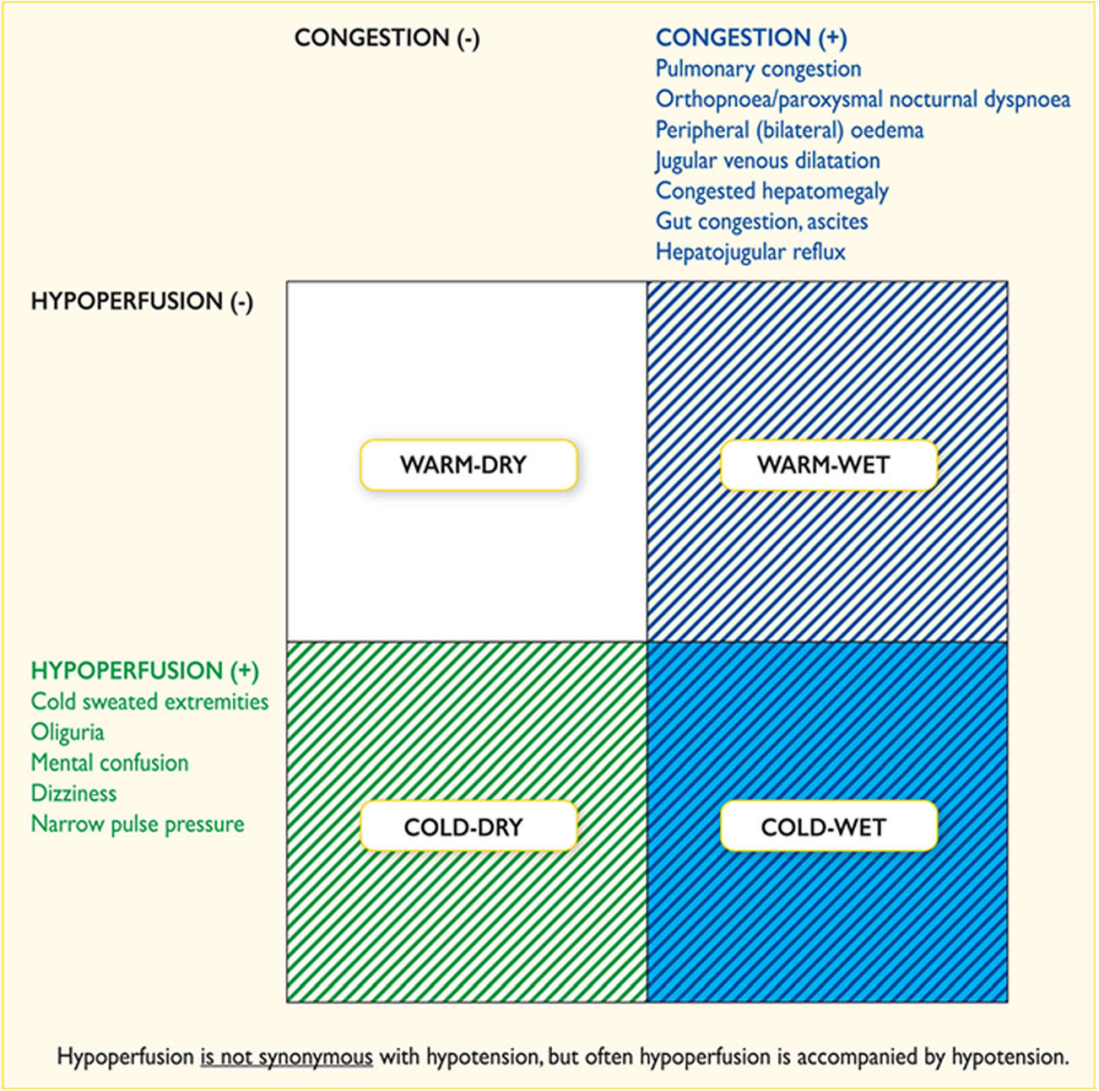
Classification of patients presenting with ADHF. Source reference [11]

Whilst there are several ways of classifying ADHF patients [6–8], they present either as decompensation of chronic HF or as a first presentation of HF (de novo). In 2008, the European Society of Cardiology (ESC) [9] classified ADHF patients into six groups based on the work of Cotter et al. [10]. Although this classification covered the various presentations of ADHF, there was much overlap within the classification in terms of the underlying pathophysiological processes which was a limitation. The most useful way of classifying ADHF is based on bedside clinical evaluation and haemodynamic profile, which is determined by the presence or absence of congestion (wet versus dry) in combination with the presence or absence of peripheral hypoperfusion (cold versus warm) [11]. This groups all patients into four groups, namely wet-warm, wet-cold, dry-warm and dry-cold or groups with equivalent names (Fig. 1). This classification was adopted for routine use in ADHF in the 2013 ACC/AHA guidelines for the management of heart failure [12] and by the ESC in their 2016 HF guidelines [13••]. This method of classification allows ADHF patients with the highest clinical risk to be identified early and effective treatment to be given promptly [14]. Once a patient is placed within the correct group, the plan of the treatment is then to consider using intravenous vasodilators and diuretics in the congested but well-perfused patients, oral evidence-based treatment in the non-congested and well-perfused patient, intravenous inotropes/vasopressors with or without diuretics and vasodilators or even mechanical circulatory support in the congested and poorly perfused patient (depending on systolic blood pressure) and finally, an intravenous fluid challenge and inotropes in the non-congested but poorly perfused patient [12, 13••].

## Diagnosis and Initial Evaluation

Diagnostic evaluation ought to be established pre-hospital if possible with continued evaluation in the emergency department (ED). Precipitants and coexisting life-threatening conditions that require urgent medical treatment (such as acute coronary syndromes, arrhythmias or pulmonary embolism) need to be identified and dealt with immediately. Furthermore, alternative causes for the patient’s signs and symptoms need to ruled out (e.g. pulmonary infection and acute renal failure). A thorough history, including the past cardiac history, eliciting the precipitating cardiac and non-cardiac causes is paramount, along with a physical examination to establish the clinical profile the patient falls into based on the presence of congestion and peripheral hypoperfusion. Following this, appropriate investigations should be performed to establish the diagnosis: resting 12-lead electrocardiogram (ECG), chest X-ray, a complete laboratory assessment (full blood count, renal function, electrolytes, liver function test, glucose, cardiac biomarkers including cardiac troponin and plasma natriuretic peptide levels as well as D-Dimer, if acute pulmonary embolism is suspected) and urgent echocardiography in haemodynamically compromised patients (cardiogenic shock, suspected structural cause, e.g. mechanical complication of acute coronary syndrome). Other early investigations may include coronary angiography, transoesophageal echocardiography and cardiac MRI scanning.

## Precipitating Factors and Underlying Causes

Whilst approximately 40–50% of ADHF admissions have no known cause [15], it is vital that a precipitating cause is identified and managed promptly. The European Society of Cardiology guideline [13••] recommends immediate identification of coexisting life-threatening clinical conditions and/or precipitants using the CHAMP acronym (acute Coronary syndrome, Hypertension emergency, Arrhythmias, acute Mechanical cause [e.g. mechanical complication of ACS, acute native or prosthetic valve regurgitation due to endocarditis, aortic dissection] and Pulmonary embolism). Other precipitating factors include non-compliance with HF medication or dietary restriction and non-cardiac triggers such as side-effects of medications (e.g. calcium-channel blocker, thiazolidinediones) and kidney injury [16]. If an underlying cause of the HF is not apparent, it is important to hunt for a cause whilst monitoring is in progress with the aim of trying to identify reversible and treatable causes. Multivariable validated risk scores may be used to assess the subsequent risk of mortality at any stage after admission [12].

## Monitoring and Treatment

### Early In-hospital Monitoring in the Emergency Department, the Coronary Care Unit or the Intensive Care Unit

Rapid diagnosis is important and initial management includes assessment for the need of oxygen therapy with or without ventilatory support. Therefore, ADHF patients should be admitted to a centre with adequate intensive care unit (ICU)/coronary care unit (CCU) facilities, where immediate cardio-respiratory support can be provided. After initial assessment, patients deemed to be high risk (cardiogenic shock, those needing ventilatory or inotropic support, high-risk acute coronary syndrome) need to be moved to the coronary care unit or the intensive care unit as appropriate. Pulmonary congestion resulting in hypoxaemia ought to be corrected with the fraction of inspired oxygen (FiO^2^) aimed to be 100%, unless contraindicated. During oxygen administration, transcutaneous oxygen saturation should be monitored. Where indicated, non-invasive positive pressure ventilation, which is especially useful in patients with COPD, ought to be commenced. Patients requiring inotropic or vasopressive support should have continuous telemetry monitoring, as they are at risk of developing arrhythmias. Those patients requiring either invasive or non-invasive ventilatory support need regular acid-base balance, pH and arterial blood gas monitoring. In case of refractory symptoms, despite appropriate management (e.g. persistent hypoperfusion), intra-arterial line and/or pulmonary artery catheterisation may be considered. Patients in cardiogenic shock who are not responding to inotropic support should be considered for mechanical circulatory support and cardiac transplantation if appropriate. A palliative strategy may be required for those where further escalation is not appropriate.

Pulmonary artery catheterisation has a limited role in patients with ADHF and should be only considered in patients (i) that are refractory to pharmacological treatment, (ii) that are persistently hypotensive, (iii) in whom LV filling pressure is uncertain or (iv) that are being considered for cardiac surgery [14]. The aim is to assess whether hypotension is related to low LV filling pressure (in which case, diuretics and vasodilators may need to be reduced and volume replacement considered) or related to high LV filling pressure/systemic vascular resistance (where use of inotropes or vasodilators will be necessary depending on the blood pressure). For those undergoing cardiac surgery, the procedure would be mainly to routinely assess pulmonary vascular resistance.

### Inpatient Monitoring on the Ward

**Fig. 2:**
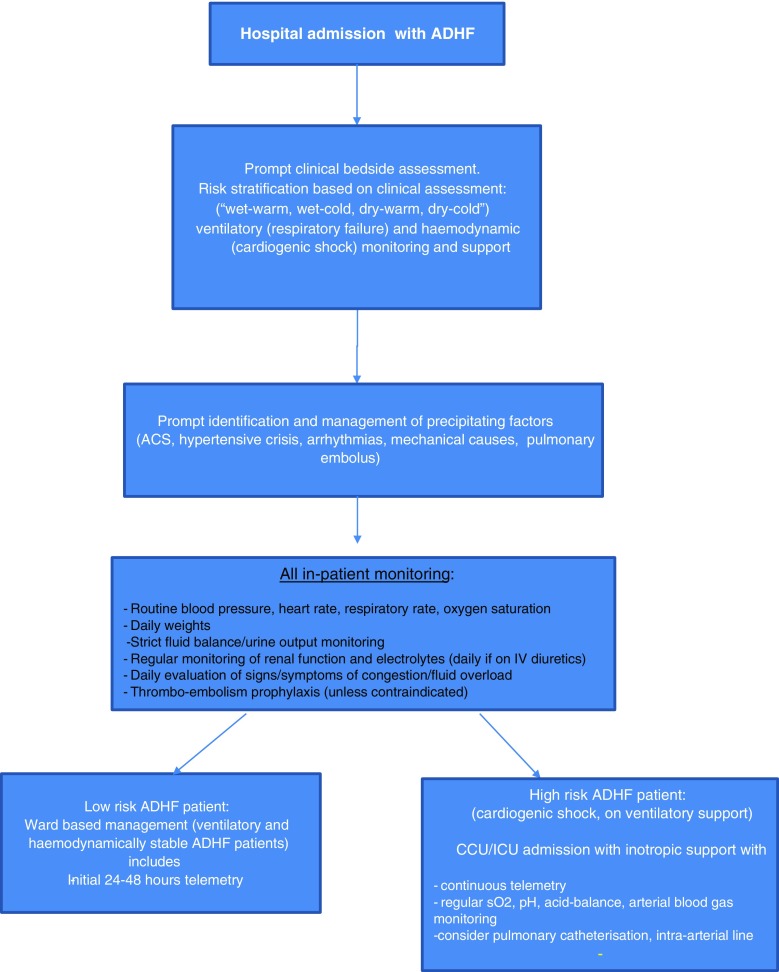
Overview of ADHF in-hospital monitoring

Low-risk ADHF patients should be monitored on the ward. As patients are at risk of hemodynamic compromise and arrhythmias, inpatient monitoring of cardio-respiratory function, including blood pressure, pulse oximetry with ECG monitoring or telemetry for at least 24 to 48 h, is vital to ensure adequate organ perfusion and oxygenation (Fig. 2). Urine output should be daily monitored, along with strict fluid balance monitoring and daily weight. However, according to the European Society of Cardiology guidelines, the routine use of urinary catheterisation is not recommended [14]. Furthermore, daily renal function and electrolytes monitoring during intravenous therapy, along with at least daily evaluation of clinical signs and symptoms of congestion, should take place. All patients should be given thromboembolism prophylaxis, unless contraindicated or already being treated with oral anticoagulation. At the time of transition from intravenous to oral diuretics, careful attention must be given to the status of congestion, supine and upright blood pressure, electrolytes and renal function. Awareness of the likely adverse effects of therapy (renal dysfunction, electrolyte abnormalities, metabolic alkalosis and symptomatic hypotension) helps in the monitoring process to prevent these. The 2017 focused update of the 2013 ACC/AHA guidelines on heart failure suggests that during a HF hospitalization, a predischarge natriuretic peptide level can be useful to establish a postdischarge prognosis [17•].

## Conclusion

In-hospital monitoring of ADHF patients is of critical importance in determining outcome. The immediate identification of the precipitating factors on presentation, including the use of the CHAMP acronym, as well as a hunt for underlying causes is vital. Intensive monitoring is of particular importance in the high-risk and really ill patients. Ample provision of haemodynamic and ventilatory support is necessary in addition to the basic monitoring available on wards. Close attention to basic observations such as blood pressure, heart rate and rhythm, oxygen saturation, fluid balance and daily weights and to daily laboratory measures such as renal function and electrolytes (with a low threshold for frequently checking haemoglobin, haematinics and acid-base balance) is the best way to tide over the acute phase and guide the patient to recovery. Continuous reassessment of the patient remains the key feature of the monitoring.
